# Minim Typing – A Rapid and Low Cost MLST Based Typing Tool for *Klebsiella pneumoniae*

**DOI:** 10.1371/journal.pone.0033530

**Published:** 2012-03-12

**Authors:** Patiyan Andersson, Steven Y. C. Tong, Jan M. Bell, John D. Turnidge, Philip M. Giffard

**Affiliations:** 1 Tropical and Emerging Infectious Diseases Division, Menzies School of Health Research, Charles Darwin University, Darwin, Northern Territory, Australia; 2 SA Pathology, Women's and Children's Hospital, Adelaide, South Australia, Australia; 3 Departments of Pathology, Paediatrics and Molecular and Biochemical Science, University of Adelaide, Adelaide, South Australia, Australia; St. Petersburg Pasteur Institute, Russian Federation

## Abstract

Here we report a single nucleotide polymorphism (SNP) based genotyping method for *Klebsiella pneumoniae* utilising high-resolution melting (HRM) analysis of fragments within the multilocus sequence typing (MLST) loci. The approach is termed mini-MLST or Minim typing and it has previously been applied to *Streptococcus pyogenes*, *Staphylococcus aureus* and *Enterococcus faecium*. Six SNPs were derived from concatenated MLST sequences on the basis of maximisation of the Simpsons Index of Diversity (*D*). DNA fragments incorporating these SNPs and predicted to be suitable for HRM analysis were designed. Using the assumption that HRM alleles are defined by G+C content, Minim typing using six fragments was predicted to provide a *D* = 0.979 against known STs. The method was tested against 202 *K. pneumoniae* using a blinded approach in which the MLST analyses were performed after the HRM analyses. The HRM-based alleles were indeed in accordance with G+C content, and the Minim typing identified known STs and flagged new STs. The *tonB* MLST locus was determined to be very diverse, and the two Minim fragments located herein contribute greatly to the resolving power. However these fragments are refractory to amplification in a minority of isolates. Therefore, we assessed the performance of two additional formats: one using only the four fragments located outside the *tonB* gene (*D* = 0.929), and the other using HRM data from these four fragments in conjunction with sequencing of the *tonB* MLST fragment (*D* = 0.995). The HRM assays were developed on the Rotorgene 6000, and the method was shown to also be robust on the LightCycler 480, allowing a 384-well high through-put format. The assay provides rapid, robust and low-cost typing with fully portable results that can directly be related to current MLST data. Minim typing in combination with molecular screening for antibiotic resistance markers can be a powerful surveillance tool kit.

## Introduction

Multilocus sequence typing (MLST) is a widely used method for determining bacterial population structures, and assigning isolates to lineages [1]. MLST is based on sequence data from standardised fragments of housekeeping genes. Usually seven fragments of approximately 450 bp are used. Unique sequences at each locus are assigned an allele number. The seven allele numbers comprise a profile that defines the sequence type (ST). Alleles and sequence types are numbered in accordance with the chronology of their discovery, and are accessible online (http://www.mlst.net/, http://www.pasteur.fr/). While MLST is very effective, it typically requires 14 separate sequencing reactions per isolate. The time and cost of this and the associated data analysis has limited the use of MLST in large-scale studies, and in the near future, MLST may not be competitive with whole genome sequencing using next generation technologies.

High-resolution melt (HRM) analysis is a low cost, and convenient technique for discriminating DNA sequence variants [2], [3], [4]. Our group has an interest in applying HRM to bacteriology [5], [6], [7], [8], [9], [10], [11], [12], [13], [14], and we have recently developed a generalised approach to bacterial genotyping approach that makes use of the MLST loci [7], [10]. The method yields data that are interpretable in terms of the MLST database of the relevant species. The central elements of this approach are: i) Sets of single nucleotide polymorphism (SNPs) that are derived by computer from concatenated MLST databases on the basis of maximisation of the Simpsons Index of Diversity (*D*), ii) PCR amplifiable fragments between approximately 50 bp and 150 bp in size that incorporate these SNPs, and also incorporate nearby SNPs that provide additional resolving power, and iii) The use of computerized keys to interpret the HRM data from analysis of these fragments in terms of the MLST database. The method is termed mini-MLST or Minim typing. Although the resolving power is somewhat less than MLST, the *D* provided by the method with respect to MLST is typically >0.98. The method cost is 10–20% of MLST, and it is single-step, closed tube. The Minim approach therefore has potential to facilitate low cost surveillance, the initial response to suspected outbreaks, and studies involving large numbers of isolates.

Minim typing has previously been applied to *Streptococcus pyogenes* [10], *Staphylococcus aureus* [7] and *Enterococcus faecium* [14]. Here we report a Minim typing method for *Klebsiella pneumoniae*.

## Materials and Methods

### Bacterial isolates

Two hundred and two clinical isolates of *K. pneumoniae* isolated between 2001 and 2010, from the SENTRY Antimicrobial Surveillance Program – Asia-Pacific (41 isolates), Australian Group for Antimicrobial Resistance (AGAR) (96 isolates), SA Pathology (Women's & Children's Hospital) Adelaide (45 isolates), and the Princess Alexandra Hospital (PAH) in Brisbane (20 isolates) were used.

### DNA isolation

DNA was purified predominantly using the automated Corbett X-tractor Gene platform (no longer available, but equivalent to the Qiagen QIAxtractor), with a small number of samples subjected to DNA purification using a DNeasy Blood and Tissue Kit (Qiagen, Doncaster Australia).

### Multilocus sequence typing

A subset of isolates from the cohort (n = 74) were selected for full MLST performed according to the protocol described by Diancourt and colleagues [15], which uses segments of seven housekeeping genes: *gapA* (glyceraldehyde 3-phosphate dehydrogenase, target sequence 450 bp), *infB* (translation initiation factor 2, target sequence 318 bp), *mdh* (malate dehydrogenase, target sequence 477 bp), *pgi* (phosphoglucose isomerase, target sequence 432 bp), *phoE* (phosphorine E, target sequence 420 bp), *rpoB* (beta-subunit of RNA polymerase, target sequence 501 bp) and *tonB* (periplasmic energy transducer, target sequence 414 bp). The MLST database for *K. pneumoniae* can be found at http://www.pasteur.fr. Population structure was visualised using eBURST v3 (http://eburst.mlst.net) [16].

### Target identification

The MinimumSNPs software [9], [17] was used to identify the most informative SNPs in the concatenated sequences downloaded from the *K. pneumoniae* MLST database. MinimumSNPs is available at http://www.ihbi.qut.edu.au/research/cells_tissue/phil_giffard/index.jsp. A Simpson's Index of Diversity (*D*) of 0.95 was used as a target point for the mining of SNPs in the MLST population. The *D*-value describes the probability that two randomly sampled STs from the population will be discriminated by the set of SNPs identified.

### Primer design

Using Primer3 (http://frodo.wi.mit.edu/primer3/) [18] and *K. pneumoniae* MGH 78578 sequence (Genbank accession number NC_009648) as template, primers were designed to flank each SNP, with the primer binding sites located in conserved regions. The secondary structure characteristics were assessed using NetPrimer (http://www.premierbiosoft.com/netprimer/index.html) in order to minimise hairpin and dimer structures. Primers used for amplification are described in Table 1 and were obtained from Sigma Aldrich (Singapore).

**Table 1:** Target SNP positions, primer information and predicted melting curves.

Gene	Target SNP	Primer name	Primer position	Sequence (5′-3′)	Amplicon length (bp)	Melting curves
*infB*	729	*infB*729-F	696–711	CCTGCCGGAAGAGTGG	50	12, 13
		*infB*729-R	730–745	TCGCGGAAACGTGGAC		
*mdh*	1197	*mdh*1197-F	1159–1178	ATTGCCGACCTGACTAAACG	58	8, 9, 10
		*mdh*1197 R	1198–1216	CTTTCGCTTCCACGACTTC		
*phoE*	2013	*phoE*2013-F	1996–2012	GAAGGGGTGGGGAGTGA	78	18, 19, 20, 21
		*phoE*2013-R	2054–2073	GGCGTTCATGTTTTTGTTGA		
*rpoB*	2227	*rpoB*2227-F	2153–2173	TGATTAACTCCCTGTCCGTGT	132	41, 42, 43, 44, 45, 46, 47
		*rpoB*2227-R	2263–2284	CGTAGTTGCCTTCTTCGATAGC		
*tonB*	2693	*tonB*2693-F	2659–2677	GTTGAACCCGAACCTGAGC	101	36, 38, 39, 40, 41, 42, 45
		*tonB*2693-R	2742–2759	GGTTTGGGCTTCGGCTTA		
*tonB*	2886	*tonB*2886-F	2783–2802	AAAAGGTTGAACAGCCGAAG	120	49, 50, 54, 55, 56, 57, 58, 59
		*tonB*2886-R	2887–2902	CCGCTGCTGTCGAGGT		

All positions are relative to the concatenated MLST sequence. The far right column describes the theoretically possible melting curves for the 6MelT system as predicted by HRMType and named according to the number of G+C residues in the alleles.

### Real-time PCR amplification and High-resolution melting

The HRM assays were developed on the Rotorgene 6000 real-time PCR platform (Corbett, Mortlake, New South Wales, Australia). This platform is no longer available, but is essentially equivalent to the Qiagen Rotor-Gene Q. Each 10 µl reaction contained: 1× Platinum® SYBR® Green qPCR SuperMix-UDG (Invitrogen Life Technologies, Mt Waverley, Australia), 0.4 µM of each primer and 1 µl of template DNA. The thermocycling parameters were: 50°C for 2 min, 95°C for 2 min, 40 cycles of [95°C for 7 s, 60°C for 7 s, and 72°C for 15 s], 95°C for 2 min and 50°C for 20 s, followed by HRM ramping from 70–95°C with fluorescence data acquisition at 0.05°C increments. The assay was also transferred and optimised for the LightCycler® 480 real-time platform (Roche, Castle Hill, New South Wales, Australia). On this platform each 10 µl reaction contained: 1× LightCycler® 480 High Resolution Melting Master (Roche Diagnostics, Castle Hill, New South Wales, Australia), with 4 mM MgCl_2_, 0.4 µM of each primer and 1 µl of template DNA. The thermocycling parameters were: 95°C for 10 min, 40 cycles of [95°C for 7 s, 61°C for 7 s, and 72°C for 15 s], 95°C for 1 min and 40°C for 1 min, followed by HRM ramping from 70–98°C, with 25 fluorescence acquisitions/°C.

### HRMType software

HRM data reflect the differences in base pairing strengths between fragments with different nucleotide sequence. In general it is much easier to discriminate alleles of SNPs that change the number of hydrogen bonds (G/C↔A/T SNPs) than to discriminate the alleles of A↔T or G↔C SNPs. To facilitate the process of predicting the combined net effect of all SNPs in each target fragment, the ‘HRMType’ software described in Richardson *et al* was used [10]. HRMType is a “do file” written using the STATA statistical analysis package (STATA 11.2, StataCorp, Texas, USA). Using concatenated and aligned MLST data, HRMType calculates predicted HRM alleles on the basis of G+C content of user defined fragments of the alignment, and converts each ST in the relevant MLST database into a predicted HRM type (MelT) on the basis of the multilocus profile of predicted HRM alleles. It also calculates ancillary information such as frequencies of predicted HRM-based genotypes, the resolving power of the HRM genotyping with respect to complete MLST analysis, and provides a key for converting between MLST and MelT data.

### Other bioinformatics analyses

Linkage analysis of MLST alleles was carried out using the LIAN software (http://adenine.biz.fh-weihenstephan.de/cgi-bin/lian/lian.cgi.pl) [19]. Comparison of the informative value offered by individual MLST alleles and various formats of the Minim assay, in relation to the MLST data was carried out using the Comparing Partitions Online tool (http://darwin.phyloviz.net/ComparingPartitions/) [20], [21], [22]. The tree drawing function available on the MLST website (http://www.pasteur.fr/recherche/genopole/PF8/mlst/Kpneumoniae.html) was used to construct a minimum spanning tree based on MLST allelic profiles.

## Results

### Method design

**Figure 1:**
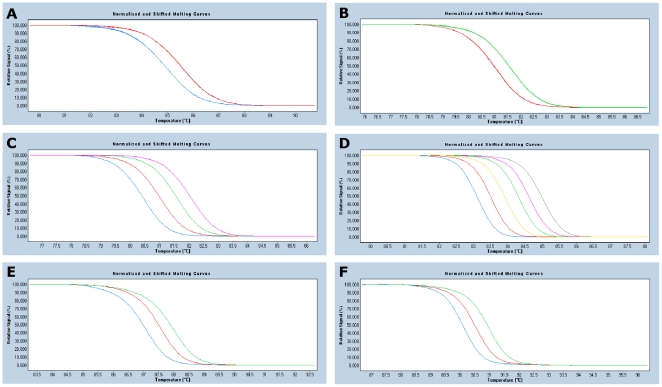
Examples of melting curves for each target region . For some target regions not all melting curves were represented among the isolates analysed in this study, therefore not all theoretically possible curves are depicted in the figure. **A**) Target region *infB*729, showing two (12, 13) of two predicted melting curves. **B**) Target region *mdh*1197, showing two (9, 10) of three predicted melting curves. **C**) Target region *pho*E2013, showing four (18, 19, 20, 21) of four predicted melting curves. **D**) Target region *rpoB*2227, showing six (41, 42 43, 44, 45, 46) of seven predicted melting curves. **E**) Target region *tonB*2693, showing three (39, 40, 41) of seven predicted melting curves. **F**) Target region *tonB*2886, showing three (54, 55, 56) of eight predicted melting curves.

In order to design a *K. pneumoniae* Minim procedure, the Minimum SNPs software was used to identify a set of six SNPs that provided as high as possible *D* value with respect to the MLST database. To maximise that probability of the SNPs forming the basis of a robust method, the choice of SNPs was confined to: i) G/C↔A/T SNPs, because in these cases, the alleles differ in their numbers of hydrogen bonds, and are therefore more likely to be resolved by HRM analysis, and ii) SNPs that are close to conserved regions that may be used for the design of PCR primer sets that will amplify fragments approximately 50–150 bp in size, which is appropriate for the Minim approach [10]. In practice this was done by iteratively performing searches for *D* optimised SNPs sets, and excluding unsuitable SNPs using the Minimum SNPs “Exclude” function, before searching again. Once a candidate set of SNP had been identified, PCR primers were designed, and shown to generate the anticipated amplification products in a small group of *K. pneumoniae* isolates (data not shown). The HRMType STATA do-file was used to predict the HRM alleles for each amplified fragment, and the resolving power of the method, as calculated against the concatenated MLST database, using the assumption that the HRM procedure assigns amplicons to alleles on the basis of G+C content. The PCR primers and amplicons are described in Table 1. An example of melting curves for each target region is shown in Figure 1. For some target regions not all melting curves were represented among the isolates used in this study. A key for translating Minim data into MLST data was assembled in Microsoft Excel using the output of the HRMType analysis and provided as supplementary data. Minim typing using six fragments was predicted to resolve the 863 STs present in the MLST database on 18 January 2012 into 148 groups or singletons, and to provide *D* = 0.979 against these STs.

### Method validation

A blinded approach was taken to determine the reliability of this method. All 202 *K. pneumoniae* isolates were subjected to Minim typing. All six Minim fragments were amplified from 182 of these. For the remaining 20 isolates, either one or both of the *tonB* derived fragments failed to amplify. Because at that time we had no isolates of known ST, the HRM data were provisionally calibrated with MLST data by inference from the frequencies of alleles obtained (for all amplified fragments, the bulk of sequences fall into one of two alleles on the basis of G+C content), whether the profiles obtained are present in our Minim-MLST conversion key, and our experience with the melting temperature (T_m_) differences that are conferred by single differences in G+C content. The provisional calibration was used to deduce Minim profiles. Of the 182 isolates, 179 yielded profiles that existed in our conversion key.

Seventy-four of these isolates were subjected to complete MLST determination. The isolates were chosen to be representative of the different MelTs obtained in our cohort. It was found that our assignment of HRM data to alleles defined on the basis of G+C content was accurate, and that accordingly the MLST and HRM data were consistent. Length variations in MLST fragments are rare (and ideally absent), and the effects of length variations on HRM data are difficult to predict, hence not considered by HRMType. However, the G+C content of the *tonB*2693 and *tonB*2886 target regions were separately calculated for all *tonB* alleles, and taken into account in the construction of the MelT↔ST conversion key. Three isolates yielded a MelT profile not in the prototype of our conversion key. The three isolates (GNB2425, N9097, and N10547) yielded new STs, in a manner fully consistent with the Minim data. Several isolates with MelTs already in the conversion key also yielded novel STs consistent with the HRM data. In total 20 novel STs were identified. Eleven of these were comprised of a novel combination of previously identified alleles, and nine isolates contained at least one previously undescribed allele. The new STs together with information regarding the relevant isolates were deposited in the *K. pneumoniae* MLST database. Complete information for all isolates is provided as supplementary data (Information S1).

### tonB is diverse: implications for assay format and data interpretation

The inability to amplify either of the *tonB* fragments in 20 (9.9%) of the isolates included in this study is potentially problematic. The *tonB* MLST fragment has 26.8% polymorphic sites, while the other six MLST fragments range from 15.2–26.6%. The ratio of non-synonymous to synonymous changes is 0.56 in the *tonB* MLST fragment, which is twice the maximum ratio seen in the other MLST loci, which range from 0.14–0.25. Twenty-eight of the 176 MLST alleles described in *tonB* by January 2012 have insertions or deletions. Linkage determinations using the LIAN software [19] of MLST allele profiles from subsets of the seven MLST loci revealed that subsets containing *tonB* have lower disequilibrium than subsets that do not include *tonB* (Table 2).

**Table 2:** Indices of association for combinations of MLST alleles with and without *tonB*, and measurements of diversity conferred by individual loci indicate that *tonB* is highly diverse and likely subjected to horizontal gene transfer.

Targets	*I_A_*
All MLST loci	0.1112
All loci excluding *tonB*	0.1279
*phoE, gapA, tonB, infB*	0.0852
*gapA, tonB, infB, mdh*	0.1002
*tonB, infB, mdh, rpoB*	0.1017
*infB, mdh, rpoB, pgi*	0.1346
*phoE, gapA, rpoB, pgi*	0.1260

	*D* against all STs
*gapA*	0.740
*infB*	0.769
*mdh*	0.786
*pgi*	0.696
*phoE*	0.931
*rpoB*	0.779
*tonB*	0.970

Taken together, these observations indicate that *tonB* is subjected to positive selection for variation and horizontal gene transfer, which in turn suggest that even on its own it may provide high genotyping resolution. These characteristics are consistent with the periplasmic location and possible immune surveillance of tonB. It is highly likely that amplification difficulties for the *tonB*-derived Minim fragments are due to primer binding site variation.

We considered alternative strategies for efficiently capturing the informative power of the *tonB* Minim fragment. The *D* values conferred by different combinations of genotyping loci were calculated (Table 3). It can be seen that the two *tonB* Minim targets increase the *D* value of the Minim method from 0.929 to 0.979, and increase the number of predicted genotypes by a factor of three. However, a protocol composed of Minim analysis of the four non-*tonB* fragments and sequencing of the *tonB* MLST fragment provides a very high *D* value of 0.995 against all STs in the database. Interestingly, as predicted, sequencing of the *tonB* MLST fragment alone also provides good resolving power (*D* = 0.970 against all STs in the database, Table 2). This is higher than any of the other MLST fragments, which range from 0.679–0.931. Therefore, several assay formats or work flows are possible. Minim typing with the four non-*tonB* fragments provides a very robust, rapid (<2 hours) and economical (<$3.00) approach to surveillance, or initial investigation of outbreaks or suspected infection control failures. If additional resolving power is required, then either the two *tonB* Minim fragments can be analysed by HRM, or the *tonB* MLST fragment sequenced. There will be rare occasions where one or both of the *tonB* Minim fragments fail to amplify. In this case, sufficient resolution may be provided by one *tonB* fragment.

**Table 3:** Discriminatory power from different combinations of markers, calculated against all STs (863 STs, *D* = 1).

	Number of types	*D*
**Hybrid method:** HRM analysis of the four non-*tonB* fragments (four fragment Minim), and sequencing of the *tonB* MLST fragment)	433	0.995
**Six fragment Minim:** HRM analysis of all six Minim fragments	148	0.979
**Four fragment Minim:** HRM analysis for the four non *tonB* Minim fragments.	43	0.929

Accordingly, three Microsoft Excel keys with drop down filters have been provided for results interpretation (Information S2). The first two of these incorporates only HRM data from four (*infB*729, *mdh*1197, *phoE*2013 and *rpoB*2227) or six (*infB*729, *mdh*1197, *phoE*2013, *rpoB*2227, *tonB*2693 and *tonB*2886) Minim fragments (for the sake of clarity these will be termed 4MelT respectively 6MelT), and the other incorporates HRM data from the four non-*tonB* Minim fragments and MLST allele data from *tonB*. The drop-down filters enable the determination of which STs are consistent with HRM alleles derived from any combination of Minim fragments, so it is straightforward to e.g. accommodate the absence of data from one or both the *tonB* Minim fragments. The files are provided as supplementary data, and regularly updated versions will be stored at www.menzies.edu.au/research/tropical-and-emerging-infectious-disease/bacterial-genotyping.

### Method Portability

The HRM analyses in this study were divided between a Rotorgene 6000 using Platinum® SYBR® Green qPCR SuperMix-UDG and a LightCycler® 480, using the LightCycler® 480 High Resolution Melting Master. Attempts to use Platinum® SYBR® Green qPCR SuperMix-UDG in the LightCycler® 480 yielded poor resolution between sequence variants (data not shown). The LightCycler® 480 also required higher MgCl_2_ concentration, which together with a saturating dye resulted in a shift of the T_m_ to a higher temperature. Twenty-nine isolates, representative of the 20 different melting curves presented in figure 1, were analysed on both platforms. Although we did observe a slightly increased variation in T_m_ of the melting curve clusters on the LightCycler® 480 compared to the Rotorgene 6000 consistent with the results of Herrmann *et al* [23], the melting curve interpretation remained robust and the tested isolates were assigned identical MelTs on both platform. The two platforms were located in different laboratories, Menzies School of Health Research in Darwin and Women's and Children's Hospital in Adelaide, further demonstrating the robustness of the assay.

### Concordance of Minim typing and MLST

The potential for these techniques to identify and discriminate *K. pneumoniae* STs of high public health significance was tested. There has been global scale dissemination of lineages or clones that belong to STs 11, 258 and 14 that express KPC/NDM carbapenemases and/or CTX-M class β-lactamases [24]. In addition, an ST23 clone or lineage associated with community acquired liver abscesses has been of considerable concern in recent years [25], [26], [27]. It can be seen in Table 4 that all three assay formats discriminate these STs, with the exception that the “four fragment” Minim method cannot discriminate ST11 and ST258. These two STs are SLVs, so the failure to discriminate them is not surprising. In most cases, STs that were not discriminated from the STs of interest by four-fragment Minim typing plus sequencing of the *tonB* MLST fragment are very closely related to the STs of interest.

**Table 4:** Power of Minim typing to identify and discriminate *K. pneumoniae* STs of particular significance.

	Resistance genes	Four fragment Minim	Six fragment Minim	Hybrid method (four fragment Minim plus *tonB* sequencing)	Relatedness of STs not discriminated by the hybrid method to the ST of interest*
ST 11	KPC	4MelT 8 (defines 47 (5.4%) of STs)	6MelT 11 (defines 9 (1.0%) of STs)	hybridMelT 138 (defines 5 (0.6%) of STs)	4 SLVs
ST 258	KPC	4MelT 8 (defines 47 (5.4%) of STs)	6MelT 72 (defines 22 (2.5%) of STs)	hybridMelT 152 (defines 8 (0.9%) of STs)	5 SLVs, 2 DLVs
ST14	KPC/NDM/CTX-M	4MelT5 (defines 143 (16.6%) of STs)	6MelT 14 (defines 55 (6.4%) of STs)	hybridMelT 51 (defines 11 (1.3%) of STs)	6 SLVs, 3 DLVs, 1 QLV
ST23	NA	4MelT 2 (defines 84 (9.7%) of STs)	6MelT 16 (defines 40 (4.6%) of STs	hybridMelT 23 (defines 17(2.0%) of STs)	9 SLVs, 5 DLVs, 2 TLV

*SLV = single locus variant, DLV = double locus variant, TLV = triple locus variant, QLV = quadruple locus variant.

An ideal typing method demarcates isolates on the basis of natural relationships. We endeavoured to determine if the groups of *K. pneumoniae* STs defined by Minim typing are concordant with the large scale *K. pneumoniae* population structure as determined by eBURST and minimum spanning tree analysis of the *K. pneumoniae* MLST allele profiles. However the global snapshot eBURST analysis described in Woodford *et al* [24] revealed a large number of singletons, a small number of clearly demarcated CCs, and a single large cluster of linked STs that form a network, characteristic of species with high frequencies of horizontal gene transmission and recombination [28], [29]. Consistent with this, the LIAN analysis revealed low linkage disequilibrium compared with other species, and within the large central network, there were many instances of double locus variants (DLVs) that were separated by many more than two steps in the eBURST diagram, and many instance of singletons that are DLVs of STs in the large interlinked complexes (data not shown). Similarly, with the minimum spanning tree, there were many instances of similar allelic profiles being widely separated on the tree (data not shown). Recombination results in populations having network rather than tree structures, resulting in no clearly separated lineages on eBURST analysis, and thus we were unable to define major lineages to test their concordance with the Minim types.

## Discussion

MLST has achieved wide acceptance in the last decade, primarily because of the complete portability of the data and the excellent on-line facilities for data storage and analysis. As a result, the MLST databases are very valuable repositories of comparative genetic information. A factor that has limited the use of MLST is the time and cost. Minim typing is designed using the relevant MLST database, and it generates data that can be easily interpreted in terms of that database. While the resolving power is less than MLST, the cost is <US$10.00 in consumables, it is performed in a single step in a generic real time PCR device, requires no specialised reagents beyond unlabelled PCR primers, and takes less then two hours. In the case of Gram-negative pathogens such as *K. pneumoniae*, the value of Minim typing may be its potential to be performed simultaneously with PCR-based assays for antibiotic resistance markers.

In the development of this method, we found that the high variability of *tonB* provided considerable resolving power, but also made the method less robust. In consequence, we developed an alternative format that includes full sequencing of the *tonB* MLST fragment. This is still much more cost effective than complete MLST determination at approximately $US15 per isolate. As expected, “four fragment” Minim typing plus complete *tonB* sequencing provides a very high D-value (0.995) with respect to the MLST database. However, there will be instances where *tonB* sequencing is not justified. The “four fragment” Minim format may be sufficient for surveillance, especially if it is performed in parallel with PCR-based resistance gene detection. Analysis of the two *tonB* fragments by HRM was successful in 90% of the isolates included in this study, and inclusion of these targets increases the *D* value from 0.926 to 0.980. In the situation where amplification of one of the *tonB* fragments fails, the other fragment provides significant resolving power, and lack of amplification constitutes a null allele that is itself informative. There was just one isolate for which we demonstrated that HRM analysis resulted in a miscall of a *tonB* MLST allele, and this was due to a deletion in the relevant *tonB* Minim fragment. We see this as a minor concern, as the relationship between the HRM data and *tonB* sequence is reproducible. This means that the genotype itself is robust, but there is a small uncertainty regarding the *de novo* inference of possible *tonB* MLST alleles from *tonB* HRM data. This is a rare phenomenon; only ten of the known *tonB* MLST alleles have indels in one or the other of the Minim fragments, and our 6MelT conversion key now incorporates this information.

We found Minim typing to be effective in discriminating and identifying STs that are of considerable clinical and public health significance. We were unable to demonstrate a high level of concordance between Minim typing and the large scale population structure. In essence this was because we were unable to define coherent lineages defined by MLST, and there is little doubt that this is because *K. pneumoniae* is a highly recombinogenic (i.e. non-clonal) species [30]. In contrast, *S. aureus* has a low frequency of recombination, and clearly defined lineages that are highly concordant with MelTs [7].

In conclusion, we have developed an HRM based method for genotyping *K. pneumoniae*. The Minim approach is rapid, simple and cost effective, and yields results that can be easily and directly interpreted in terms of MLST data. Although less discriminatory than MLST, its strength lies in being able to rapidly and cost-effectively screen large sample sets. It is particularly suitable for combining with PCR-based resistance gene detection, and used in this way would be an effective approach for surveillance for the arrival of important antibiotic resistant clones.

## Supporting Information

Information S1 ***Klebsiella pneumoniae*** **sample data.**
(XLS)

Information S2 **MelT↔ST conversion key for 863 STs.**
(XLS)
